# Whole-exome sequencing identifies distinct genomic aberrations in eccrine porocarcinomas and poromas

**DOI:** 10.1186/s13023-025-03586-7

**Published:** 2025-02-13

**Authors:** Maya Puttonen, Henrikki Almusa, Tom Böhling, Virve Koljonen, Harri Sihto

**Affiliations:** 1 Department of Pathology, University of Helsinki and Helsinki University Hospital, P.O Box 63, 00014 Helsinki, Finland; 2 Institute for Molecular Medicine Finland, FIMM, University of Helsinki, Helsinki, Finland; 3 Department of Plastic Surgery, University of Helsinki and Helsinki University Hospital, Helsinki, Finland

**Keywords:** Eccrine porocarcinoma, Eccrine poroma, Whole-exome sequencing, Skin cancer

## Abstract

**Background:**

Eccrine porocarcinoma (EPC) is a rare malignant skin tumor arising from the eccrine gland. Investigations into the genomic landscape of EPC have uncovered potential drivers of its development and progression. However, there is limited information on the discrepancies between EPC and its benign counterpart, eccrine poroma (EP).

**Methods:**

Formalin-fixed paraffin-embedded (FFPE) samples from 15 EPCs and 5 EPs were retrieved from Helsinki Biobank and Finnish Clinical Biobank Tampere. One EPC was found to be digital papillary adenocarcinoma in review of diagnoses. Whole-exome sequencing was used to conduct a comprehensive analysis to elucidate the genomic features of EPCs and EPs.

**Results:**

There was general heterogeneity within EPCs and EPs, with discrepancies such as exclusive *TP53*, *NCOR1*, and *CDKN2A* mutations in EPCs and a higher mutational load in EPCs than in EPs. Furthermore, we identified alterations in pathways associated with cell adhesion and the extracellular matrix in EPCs, while pathways associated with ketone body and amino acid metabolism were altered in EPs. The MAPK and Ras signaling pathways were enriched in genes mutated only in EPCs.

**Conclusions:**

EPCs and EPs are generally heterogeneous tumor entities with a few distinct discrepancies from each other. The findings from this study emphasize the need to further verify the roles of disrupted genes and pathways in the initiation and progression of EPCs and EPs.

**Supplementary Information:**

The online version contains supplementary material available at 10.1186/s13023-025-03586-7.

## Background

Eccrine porocarcinoma (EPC) is a rare malignant skin tumor arising from the acrosyringium, the intraepidermal spiral duct of the eccrine gland [1]. The presentation of EPC and EP is highly variable, making diagnosis challenging. EPC and EP typically appear as solitary erythematous, skin-colored, or violaceous nodules or papules, which may be ulcerated or prone to bleeding [2–4]. EPC mostly affects people in the seventh to ninth decades [5, 6]. The benign counterpart of EPC, eccrine poroma (EP), usually affects middle-aged to elderly people [3]. Although the etiology of EPC is still not fully understood, chronic immunodeficiency may be a risk factor [5]. Approximately 18–50% of EPCs originate from EP [7–9], with chronic ultraviolet exposure and immunosuppression hypothesized to play a role in malignant transformation [6]. From 2007 to 2017, the age-standardized rate of EPCs was 0.06 and 0.04 per 100 000 person-years for men and women in Finland, respectively [2]. The corresponding rate was 0.25 per 100 000 person-years from 2013 to 2018 in the United Kingdom [10] and 0.05 per 100 000 person-years from 2000 to 2018 in the USA [11]. Although the histopathologic features of EPC are varied, some common characteristics include poromatous basaloid cells, nuclear atypia, increased mitotic activity, and necrosis. Additionally, the tumor cells frequently show ductal differentiation [12]. Although the histopathologic features of EP are varied, common features include the presence of poroid or cuticular cells [3].

The primary treatment for EP and early-stage EPC is surgical resection. Mohs micrographic surgery is increasingly used due to its lower recurrence rate compared with conventional wide local excision [12]. Currently, there are no standardized therapy regimens for patients with EPC after surgery. Metastasis is associated with a high mortality rate [7], necessitating investigations for novel therapeutic targets.

Knowledge on the genomic landscape of EPC and EP has recently increased due to accumulating research findings. Involvement of *TP53* tumor suppressor has been suggested since Harms et al. reported recurrent mutations of *TP53* in EPCs [13]. Furthermore, a high mutation frequency of *TP53* in EPCs has been reported in multiple publications [14–18]. Bosic et al. reported *TP53* mutations exclusively in EPCs [14]. *TP53*, a well-known tumor suppressor gene, has been implicated in more than 50% of all cancers [19]. *TP53* exhibits a multifaceted role as a cell-cycle regulator, apoptosis inducer, transcription factor, and transcription repressor [20]. Other notable candidate driver genes in EPCs include *EGFR* [13, 18, 21], *CDKN2A* [13, 14, 16–18, 21], and *PIK3CA* [22, 23]. *EGFR* encodes the epidermal growth factor receptor, which functions as an intrinsic tyrosine kinase. Aberrations in *EGFR* are implicated in various cancers, with amplification being the most common. This has been reported in glioblastoma multiforme [24], breast carcinomas [25], non-small cell lung cancers [26], and colorectal cancers [27]. Loss of the tumor suppressor gene *CDKN2A* is a critical event in the tumorigenesis and progression of various cancer types, including melanoma [28–30]. The encoded protein p16 acts as an inducer of cell cycle arrest in G1 and G2 phases by inhibiting CDK4/6 [31, 32], while p14 functions as a stabilizer of p53 [33]. The phosphatidylinositol 3-kinase (PI3K) signaling pathway plays a crucial role in regulating cell growth, proliferation, motility, angiogenesis, and survival [22]. Mutations in *PIK3CA* are frequently observed in various cancers, particularly in breast cancer [34, 35]. *HRAS* mutations have been reported at similar frequencies in both EPCs and EPs [13, 14]. RAS proteins regulate proliferation, differentiation, cell adhesion, and cell migration through activation of the mitogen-activated protein (MAP) kinase pathway [36]. There is also a growing interest in fusion genes following the recent discovery of cell-transforming *YAP-MAML2* and *YAP1-NUTM1* fusions and fusions involving the *PAK2* gene in EPCs and EPs [37]. Genomic instability resulting from changes in the homologous recombination repair pathway has also been implicated [17]. Multiple studies have reported a high overall mutation rate attributed to UV-induced mutational signatures [13, 14, 17, 18]. However, there is a lack of studies investigating possible driver mutations that exclusively define the development of the malignancy or malignant transformation from EP to EPC at the genomic level.

In the present study, we sought to describe the genetic features of EPCs and EPs using whole-exome sequencing (WES). To our knowledge, this is the first WES study with multiple EPCs and EPs.

## Methods

### Patient samples

Following approval from the institutional ethics committee of Helsinki University Central Hospital [HUS/358/2018], formalin-fixed paraffin-embedded (FFPE) samples from 13 EPCs and 5 EPs diagnosed in 1987–2013, along with corresponding clinical data, were collected from the Helsinki biobank. Two of the EPC tissue samples were recurrences from the former excision; the others were primary tumors. DNA was extracted from the FFPE samples. Additionally, two EPC FFPE samples were collected from the Finnish Clinical Biobank Tampere [BB2018-006]. The diagnoses of 15 EPCs and 5 EPs were reviewed by an experienced pathologist (Tom Böhling). One sample collected as an EPC was found to be a digital papillary adenocarcinoma (DPA). The DPA sample was included in our study due to its rarity and the prospect of enriching our study with novel insights.

### DNA extraction from FFPE samples

For DNA extraction, two or three 1-mm core punches were taken from the tumor area of the FFPE blocks with an automated tissue microarrayer (TMA Grand Master, 3DHISTECH Ltd., Budapest, Hungary). Genomic DNA was extracted using a QIAsymphony DSP DNA mini kit (catalog number 937236, Qiagen GmbH, Hilden, Germany) and a QIAsymphony instrument following the manufacturer’s protocols.

### Sample selection for exome sequencing

The mean concentration of DNA samples collected was 28.4 ng/µl (range 0–98.9 ng/µl). One EPC DNA sample was excluded from WES due to low DNA yield.

### Whole-exome sequencing and bioinformatics

DNA samples from 13 EPCs, 5 EPs, and one DPA were subjected to WES at the sequencing unit of the Institute of Molecular Medicine Finland. A total of 50 ng of DNA per sample was processed according to the Twist Human Core Exome EF Multiplex Complete kit manual (catalog number 100252, Twist Bioscience, San Francisco, CA, USA). 4 µl of 15 µM unique dual-index oligos with unique molecular barcodes (Integrated DNA Technologies, Coralville, IA, USA) were used for ligation. Library quantification and quality check were performed using LabChip GX Touch HT High Sensitivity assay (catalog number CLS760672, Revvity, Waltham, MA, USA). Libraries were pooled into 7- to 8-plex reactions according to concentration. Exome enrichment was performed using Twist Comprehensive Exome probes. The captured library pools were quantified for sequencing using KAPA Library Quantification Kit (catalog number KK4824, KAPA Biosystems, Wilmington, MA, USA) and LabChip GX Touch HT High Sensitivity assay. WES was performed with an Illumina NovaSeq system using a S4 flow cell with lane divider (Illumina, San Diego, CA, USA) and v1.5 chemistry. The read length for the paired-end run was 2 × 151 bp.

The protein-coding mutations were filtered through the following steps: the WES samples were analyzed with Illumina Dragen v3.8.4 using the GRCh38 reference genome. Reads were trimmed and sample analysis including variant calling was performed with tumor-only options. A comprehensive description of Dragen is available in a recent study [38]. After analysis, the called variants were annotated using Annovar [39]. Variants were further filtered by applying the following criteria: (1) variants were within the target; (2) somatic variants in Dragen evaluation; (3) unless splice variants are in question, variants were not synonymous; (4) variants were not outside of genes or within introns; (5) population frequency in gnomAD or in the gnomAD European (Finnish) population ≤ 0.05; (6) only PASS mutations; (7) REVEL [40] ≥ 0.5 for nonsynonymous single nucleotide variants (SNVs); and (8) “NA” (not applicable) for variants in genes with no REVEL values, stop-gains, insertions, and deletions, as REVEL does not apply a value for these variants. All variants from each sample that passed the filtering were provided in a text file format uploaded to Zenodo (DOI: https://doi.org/10.5281/zenodo.10701230). The codes used in this study are available on GitHub (https://github.com/Rare-Cancers-Research-Group/Porocarcinoma).

### Mutational signature analysis

SigneR R package was used to determine the appropriate number of signatures that sufficiently represent our dataset [41]. Based on the analysis, the optimal number of signatures was 5 (see Additional file 1). Five de novo mutational signatures were extracted from a 96-class mutational context using the “decipherMutationalProcesses” function of MutSignatures R package [42].

The de novo signatures were then matched with COSMIC signatures v.3.3 [43] using the “matchSignatures” function of MutSignatures R package. All COSMIC signatures with a proposed etiology of “unknown” with no other annotations or “possible sequencing artefacts” were excluded from the matching analysis.

The samples were then subjected to clustering based on the signature components using ClustVis 2.0 [44].

### Genetic overview of genes with previously reported mutations in EPC and EP

A list of oncogenes, previously identified as recurrently mutated, was selected for analysis (Additional file 2) to investigate their mutation frequency in our series. The selection was based on previously reported sequencing studies on EPC and EP [13–17]. The data from studies using Sanger sequencing and those based on a single sample were excluded from the selection, except for the study by Thibodeau et al. [21], which performed whole genome sequencing and RNA sequencing on a single EPC sample and reported amplification and elevated expression of GSK3B.

### Filtering of protein-coding mutations

To explore potential novel driver genes and pathways, the mutated genes meeting the aforementioned filtering criteria underwent additional refinement steps. Genes that were recurrently mutated only in EPCs (6/13 or more), only in EPs (2/5 or more), and in both 6/13 or more EPCs and 2/5 or more EPs (≥ 40% of both tumor types) were selected into separate lists. Genes on these lists were further filtered using the Genome Aggregation Database (gnomAD) v2.1.1 [45]. The gnomAD database provides information on predicted gene pathogenicity. Z-scores indicate tolerance to missense and synonymous mutations, with higher scores reflecting greater intolerance. Conversely, pLI scores (ranging from 0 to 1) predict tolerance to loss-of-function variants, with pLI ≥ 0.9 indicating high intolerance. Following gnomAD guidelines, a cutoff pLI score ≥ 0.9 and missense Z-score ≥ 3.1 were applied [45]. The selected genes were then analyzed using the EnrichR analysis tool (accessed on 7 April 2024) [46–48] to explore possible driver pathways. Four pathway databases, namely Bioplanet 2019, KEGG 2021 Human, WikiPathway 2023 Human, and Reactome 2022, were utilized for this analysis.

### Statistical analysis

The association of the non-continuous parameters with the continuous parameters was evaluated using the Mann–Whitney *U* test. The association between the non-continuous parameters was evaluated using the Pearson χ^2^ test or the Fisher’s test as appropriate. Statistical analysis was performed with SPSS software (IBM SPSS Statistics for Windows ver.29). *P* values < 0.05 were considered statistically significant.

## Results

### Patient overview

Patient and tumor data are summarized in Table 1. There was no statistically significant association between EPC or EP and age (*p* = 0.615), tumor size (*p* = 0.080), sex (*p* = 0.314), or tumor location (*p* = 0.519).

**Table 1:** Patient data of sequenced samples

Characteristics	EPC (n = 13)	EP (n = 5)	DPA (n = 1)
*Mean age at first diagnosis (range), years*	71 (19–89)	72 (61–87)	22
*Sex, n (%)*			
Male	6 (46)	4 (80)	1
Female	7 (54)	1 (20)	0
*Tumor diameter (range), mm*	48.0 (5–150)	17.5 (10–35)	5
N.A	3	1	
*Metastasis at diagnosis, n (%)*	3 (23)	0	0
N.A	2		
*Survival, n (%)*			
alive	8 (62)	3 (60)	
deceased	5 (38)	2 (40)	
N.A	0	0	1
*Follow-up time (range), months*	128 (15–254)	152 (18–333)	
N.A	1	0	1
*Location, n (%)*			
Head and neck	4 (31)	2 (50)	0
Trunk	4 (31)	1 (25)	0
Extremities	5 (38)	1 (25)	1
N.A		1	0

N.A., not available; EPC, eccrine porocarcinoma; EP, eccrine poroma; DPA, digital papillary adenocarcinoma

### Overview of WES data

A mean read depth of 86.0x (range 30.8–178.6) was achieved. A total of 94.8% (range 73.3–97.5) of the targeted regions were covered to at least 20x. The mean total of aligned reads was 50.9 million (range 19.8–104.1 million). The following rules were applied for counting reads: (1) duplicate reads were ignored; (2) overlapping mates were double-counted; (3) reads with MAPQ > 0 were included. MAPQ = 0 reads were filtered.

A mean of 112 212 (range 44 772–237 956) variants were called, from which 94.4% (range 92.9–95.5) were SNVs. The mean total of variants was larger in EPCs (121 078, range 44 772–237 956) compared with EPs (91 447, range 67 695–118 807). After applying the seven filters mentioned above, the mean number of variants was reduced to 3559 (range 199–8503) for EPCs and 2044 (range 1146–2697) for EPs. In the DPA sample, the total number of variants was 100 793, which was reduced to 2252 after filtering. The following analyses were conducted with these filtered datasets.

### Mutational signatures of EPC and EP

**Fig. 1:**
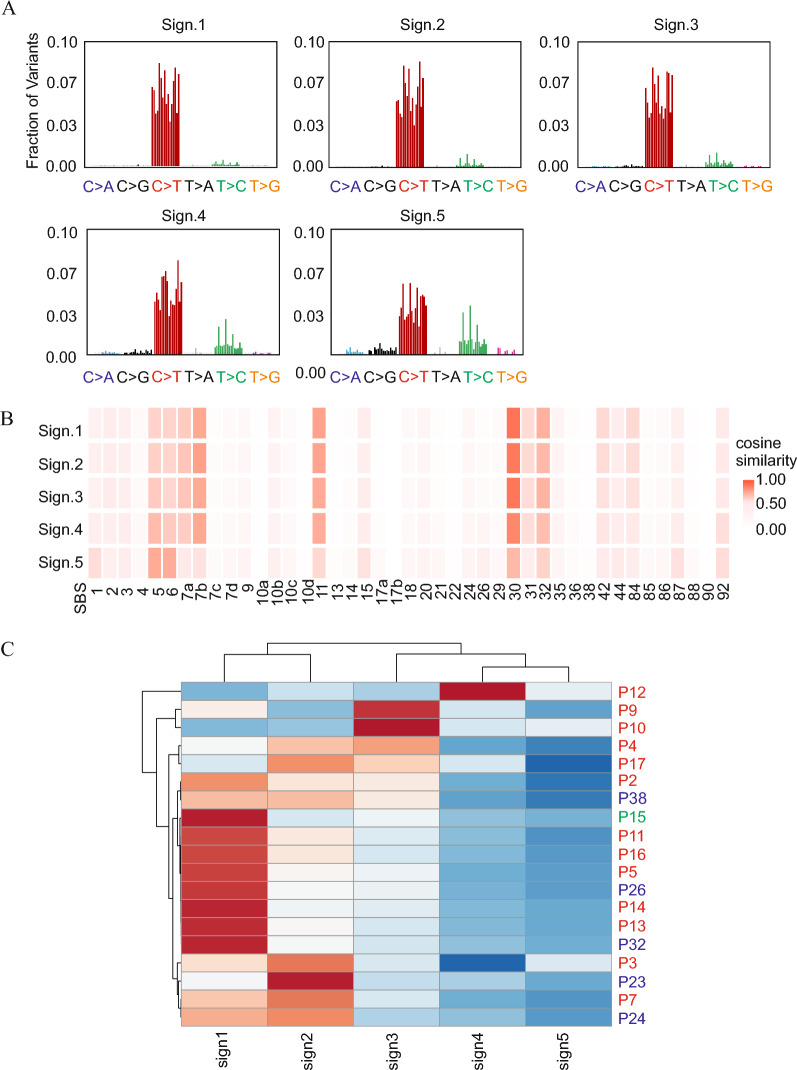
Mutational signature analysis. **A** Five de novo signatures were identified. **B** Comparison of five de novo signatures to COSMIC single-base substitutions database. **C** Clustering of the samples based on signature components. EPC samples are indicated in red letters; EP samples are indicated in blue letters; and DPA sample is indicated in green

Five de novo mutational signatures, labeled “sign1-5” were identified with a notable prevalence of C > T mutations (Fig. 1A). Sign 4 had more T > C and C > G mutations than signs 1–3, while sign 5 had even fewer C > T mutations and more T > C, C > G, C > A, and T > G mutations. When compared with the COSMIC v.3.3 single-base substitutions (SBS) database, SBS 7a and 7b UV-signatures exhibited significant similarity to signs 1–4. Both UV signatures had some similarity to sign 5. SBS 30 signature (base excision repair deficiency) was highly similar to all de novo signatures. Additionally, signatures such as SBS11 (alkylating agents) and SBS32 (azathioprine exposure) exhibited similarities especially with sign 1–4. SBS6 (defective DNA mismatch repair) and SBS5 (clock-like signature) resembled sign 5 (Fig. 1B).

The samples were clustered based on the signature components (Fig. 1C). Among the five EPs, two (P26 and P32) had a high weight for sign 1, one (P38) had relatively high weights for both sign 1 and sign 2, and two (P23 and P24) had a high weight for sign 2. DPA had a high weight for sign 1. The other samples were EPCs. Notably, there were no discernible differences in distribution patterns between EPs and EPCs.

### Genetic overview of genes with previously reported mutations in EPC and EP

**Fig. 2:**
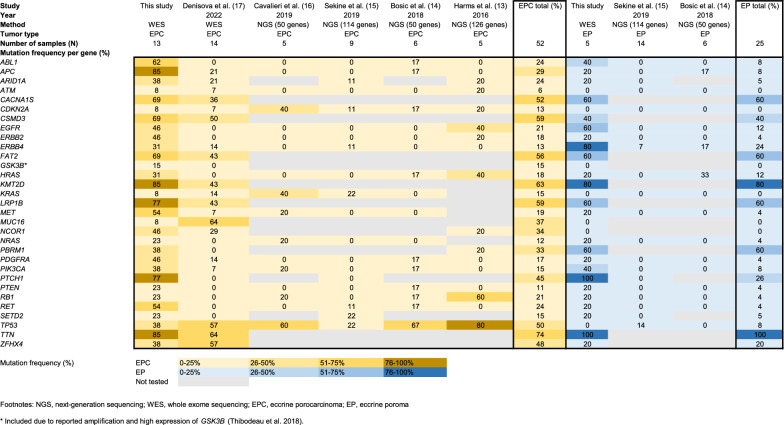
Mutation frequency of genes per patient. The heat map displays the frequency of mutations for each gene across different tumor types (EPC, orange; EP, blue). N, number; NGS, next generation sequencing; WES, whole exome sequencing; * Included due to reported amplification and high expression of GSK3B [21]

The mutation frequency analysis of the curated genes (Additional file 2) identified exclusive mutations of *TP53* in EPCs (38%) (Fig. 2). *TP53* stop-gain SNVs were identified in three EPC samples, and nonsynonymous SNVs were identified in four EPC samples, with no indels observed. Other mutations exclusively observed in EPCs included *NCOR1* (46%), *GSK3B* (15%), *ATM* (8%), *CDKN2A* (8%), *KRAS* (8%), and *MUC16* (8%). *NCOR1* contained stop-gain mutations in two, nonsynonymous SNVs in three, and frameshift insertions in two samples. One sample harbored a *CDKN2A* nonsynonymous SNV. One EPC sample had four *MUC16* stop-gain mutations. All mutations detected in *GSK3B* were nonsynonymous SNVs.

The mutation frequency of *APC* was significantly higher in EPCs (85%) than in EPs (20%). None of the selected genes had exclusive mutations in EPs, although *ERBB4* had a higher mutation frequency in EPs (80%) than in EPCs (31%). Most selected genes had similar mutation frequencies between EPCs and EPs. The DPA harbored mutations in *ABL1*, *APC*, *CDKN2A*, *ERBB4*, *FAT2*, *KMT2D*, *LRP1B*, *NCOR1*, *PDGFRA*, *PTCH1*, *RET*, *SETD2*, *TP53*, and *TTN*.

### Association of WES mutations with biological pathways

The protein-coding mutations that passed the aforementioned filtering (uploaded to Zenodo, DOI: https://doi.org/10.5281/zenodo.10701230) were further examined using four pathway databases (Bioplanet 2019, KEGG 2021 Human, WikiPathway 2023 Human, Reactome 2022) to explore possible driver pathways.

The top 10 terms enriched in the genes mutated exclusively in EPCs are shown in Table 2, and those enriched exclusively in EPs are shown in Table 3. All the statistically significant terms can be found in Additional file 3. Among the genes mutated only in EPCs, pathways associated with cell adhesion, extracellular matrix, fatty acid metabolism, microRNA, and PDGF ranked at the top. Ras and MAPK signaling pathways were also on the list of statistically significant terms. In the list of genes mutated only in EPs, pathways associated with ketone body metabolism, the tricarboxylic acid cycle, and amino acid metabolism ranked at the top.

**Table 2:** Top 10 pathway terms in EPCs

Database	Term	*P* value	Adjusted *P* value
BioPlanet	Axon guidance	4.3553914739453243E-4	0.09401196290991816
	Ion transport by P-type ATPases	4.6168069209550543E-4	0.09401196290991816
	L1CAM interactions	6.623917204297982E-4	0.09401196290991816
	Integrin-mediated cell adhesion	8.356618925326059E-4	0.09401196290991816
	Developmental biology	0.0019347762203373161	0.1479286652719452
	Ion channel transport	0.0021579633736902594	0.1479286652719452
	Actin cytoskeleton regulation	0.0026381437319520913	0.1479286652719452
	Focal adhesion	0.0030076855364787763	0.1479286652719452
	Integrin signaling pathway	0.0041414744602510985	0.1479286652719452
	Mitochondrial pathway of apoptosis: caspases	0.004179942373133494	0.1479286652719452
Reactome	Extracellular Matrix Organization R-HSA-1474244	3.053340896181574E-5	0.014014834713473425
	Degradation Of Extracellular Matrix R-HSA-1474228	9.611779645525344E-5	0.022059034286480664
	L1CAM Interactions R-HSA-373760	8.047871293697965E-4	0.09901201929697943
	Intra-Golgi And Retrograde Golgi-to-ER Traffic R-HSA-6811442	9.953675758627512E-4	0.09901201929697943
	IRF3-mediated Induction Of Type I IFN R-HSA-3270619	0.0013193116016079933	0.09901201929697943
	Ion Transport By P-type ATPases R-HSA-936837	0.0014389155203574627	0.09901201929697943
	Axon Guidance R-HSA-422475	0.001509987222394022	0.09901201929697943
	STING Mediated Induction Of Host Immune Responses R-HSA-1834941	0.0020131762554888555	0.10457112646089149
	Nervous System Development R-HSA-9675108	0.002050414244331206	0.10457112646089149
	Integrin Cell Surface Interactions R-HSA-216083	0.002702767860978328	0.12405704481890525
KEGG	Transcriptional misregulation in cancer	0.001293923047887993	0.11311594676079074
	MicroRNAs in cancer	0.0019505201670179141	0.11311594676079074
	Regulation of actin cytoskeleton	0.002257516615221331	0.11311594676079074
	Retinol metabolism	0.002942286284866666	0.11311594676079074
	Phospholipase D signaling pathway	0.003512917601266793	0.11311594676079074
	Gap junction	0.006066892682017483	0.1627949536341358
	Pathogenic Escherichia coli infection	0.009547362995081311	0.1954174856669846
	Focal adhesion	0.010223815684022936	0.1954174856669846
	Leukocyte transendothelial migration	0.01229954311583523	0.1954174856669846
	Human papillomavirus infection	0.012831613264285587	0.1954174856669846
Wikipathway	Vitamin A And Carotenoid Metabolism WP716	7.812701588013005E-4	0.0932415863194107
	Integrin Mediated Cell Adhesion WP185	8.673635936689367E-4	0.0932415863194107
	Focal Adhesion WP306	0.0015155273303906114	0.10861279201132716
	Congenital Generalized Lipodystrophy WP5101	0.002552851320063413	0.12234906640991443
	Fatty Acid Biosynthesis WP357	0.0038125656163165316	0.12234906640991443
	Alzheimer 39 S Disease And miRNA Effects WP2059	0.004788343677102684	0.12234906640991443
	Alzheimer 39 S Disease WP5124	0.004788343677102684	0.12234906640991443
	DNA IR Damage And Cellular Response Via ATR WP4016	0.004817142031573357	0.12234906640991443
	Lipid Metabolism Pathway WP3965	0.006574607345419787	0.12234906640991443
	PDGFR Beta Pathway WP3972	0.006574607345419787	0.12234906640991443

**Table 3:** Top 10 pathway terms in EPs

Database	Term	*P* value	Adjusted *P* value
BioPlanet	Pyruvate dehydrogenase (PDH) complex regulation	0.0015499404872296638	0.2986966557537662
	Glycine, serine and threonine metabolism	0.010922659437504534	0.2986966557537662
	Signaling events regulated by Ret tyrosine kinase	0.01595661006315771	0.2986966557537662
	Tricarboxylic acid (TCA) cycle	0.01595661006315771	0.2986966557537662
	Amino acid metabolism	0.01608275049968628	0.2986966557537662
	Pyruvate metabolism and citric acid (TCA) cycle	0.0167428723048413	0.2986966557537662
	Gastrin-CREB signaling pathway via PKC and MAPK	0.019261888040957735	0.2986966557537662
	Valine, leucine and isoleucine degradation	0.020048578419772704	0.2986966557537662
	Transfer RNA aminoacylation	0.02179538513328187	0.2986966557537662
	G-protein signaling through tubby proteins	0.024508443549026968	0.2986966557537662
Reactome	RET Signaling R-HSA-8853659	0.0167428723048413	0.505515062511835
	tRNA Modification In Nucleus And Cytosol R-HSA-6782315	0.01836394521075394	0.505515062511835
	SUMOylation Of Transcription Cofactors R-HSA-3899300	0.019198403941144727	0.505515062511835
	G Alpha (Q) Signaling Events R-HSA-416476	0.021148819776543717	0.505515062511835
	Signaling By Membrane-Tethered Fusions Of PDGFRA Or PDGFRB R-HSA-9673768	0.024508443549026968	0.505515062511835
	Sodium-coupled Phosphate Cotransporters R-HSA-427652	0.024508443549026968	0.505515062511835
	Regulation Of HMOX1 Expression And Activity R-HSA-9707587	0.024508443549026968	0.505515062511835
	Synthesis Of Wybutosine At G37 Of tRNA(Phe) R-HSA-6782861	0.02933831724779352	0.505515062511835
	Synthesis Of Epoxy (EET) And Dihydroxyeicosatrienoic Acids (DHET) R-HSA-2142670	0.038927163698028566	0.505515062511835
	CREB3 Factors Activate Genes R-HSA-8874211	0.038927163698028566	0.505515062511835
KEGG	Valine, leucine and isoleucine degradation	0.0017312869110559788	0.19390413403826962
	Glycine, serine and threonine metabolism	0.0167428723048413	0.6779113116897953
	Cysteine and methionine metabolism	0.025470075176232815	0.6779113116897953
	Sulfur relay system	0.038927163698028566	0.6779113116897953
	Lysine degradation	0.03896577802593897	0.6779113116897953
	Cortisol synthesis and secretion	0.04123791892332742	0.6779113116897953
	Sulfur metabolism	0.048422236549271094	0.6779113116897953
	Synthesis and degradation of ketone bodies	0.048422236549271094	0.6779113116897953
	Gap junction	0.07059348565548233	0.6847458704421556
	Selenocompound metabolism	0.08092953799668379	0.6847458704421556
Wikipathway	Hereditary Leiomyomatosis And Renal Cell Carcinoma Pathway WP4206	0.004348408989517873	0.3135156768282677
	Metabolic Pathways Of Fibroblasts WP5312	0.010922659437504534	0.3135156768282677
	Kisspeptin Kisspeptin Receptor System In The Ovary WP4871	0.01595661006315771	0.3135156768282677
	Pancreatic Cancer Subtypes WP5390	0.023602999269332906	0.3135156768282677
	Disorders In Ketone Body Synthesis WP5175	0.024508443549026968	0.3135156768282677
	Ketone Bodies Synthesis And Degradation WP311	0.024508443549026968	0.3135156768282677
	mRNA Protein And Metabolite Inducation Pathway By Cyclosporin A WP3953	0.03414451913348685	0.3135156768282677
	TYROBP Causal Network In Microglia WP3945	0.03674363191396941	0.3135156768282677
	TAR Syndrome WP5362	0.037848381018999164	0.3135156768282677
	Glucose Metabolism In Triple Negative Breast Cancer Cells WP5211	0.038927163698028566	0.3135156768282677

## Discussion

In this study, we sought to explore the exon characteristics of EPCs and EPs to provide novel insights into potential driver mutations and pathways and to examine the genetic aspects that differentiate EPCs from EPs.

WES revealed a notable prevalence of UV-induced mutational signatures in both EPCs and EPs, which is consistent with previous findings on UV-induced mutations in skin malignancies [13, 14, 17, 18]. Additionally, our de novo signatures exhibited a striking similarity to the SBS30 signature associated with base excision repair deficiency, which may be attributed to inactivating mutations in *NTHL1* [49]. Nevertheless, *NTHL1* mutations were only observed in a limited number of cases (2 EPCs and 1 EP) within our cohort, raising questions regarding the general association of SBS30 with our study population. Notably, some samples had a higher weight of a clock-like signature, consistent with the generally advanced age of onset in EPCs [5, 6]. Additionally, SBS6 (defective DNA mismatch repair) exhibited similarities with all de novo signatures, indicating potential microsatellite instability in both EPCs and EPs.

Among the previously reported genes with mutations in EPs and EPCs, exclusive mutations in EPCs were observed in *NCOR1* (46%), *TP53* (38%), *ATM* (8%), *CDKN2A* (8%), *GSK3B* (15%), *KRAS* (8%), and *MUC16* (8%), while no genes exhibited exclusive mutations in EPs. Many genes, including *PTCH1, KMT2D, LRP1B, ABL1, CACNA1S, CSMD3, FAT2, PBRM1, PIK3CA, ARID3A, EGFR, ERBB2, HRAS, ZFHX4*, and *TTN*, were recurrently mutated in both EPCs and EPs.

Nuclear receptor corepressor 1 (*NCOR1*) was among the most mutated genes. The gene was recurrently mutated in EPCs across all previous studies that examined it. However, no prior studies have investigated *NCOR1* mutations in EPs. *NCOR1* is a transcriptional corepressor that represses the tolerogenic program in dendritic cells, and its depletion leads to upregulation of tolerogenic genes [50]. In bladder cancer, *NCOR1* alterations correlate with the efficacy of immune-checkpoint blockade therapy, indicating its potential as an immunotherapy biomarker [51]. Given the successful treatment outcomes with immune-checkpoint inhibitors and imiquimod in a few EPC cases [52, 53], further investigation into the predictive value of *NCOR1* for immunotherapy response in EPC patients and subsequent clinical trials are warranted. *CDKN2A* was mutated exclusively in EPCs in all studies that examined the gene. Additionally, *ATM* and *KRAS* mutations were exclusively found in EPCs in other studies as well.

Pathway analyses revealed a significant enrichment of terms associated with the extracellular matrix (ECM) and cell adhesion in EPCs. ECM remodeling plays a significant role in the development, progression, invasion, and therapeutic resistance of skin cancers [54–56]. Alterations in cell adhesion molecules, such as integrins, cadherins, and selectins, have also been widely reported in skin cancers [57, 58]. The stiffness of the ECM caused by remodeling prevents the penetration of many antitumor treatments, and treatments targeting ECM elements, such as collagen and hyaluronic acid, are already in clinical use [59]. The enrichment of terms associated with ECM and cell adhesion was not seen in the genes mutated recurrently only in EPs, in which terms associated with metabolism were enriched.

Although not ranked in the top 10 list of terms, the MAPK and Ras signaling pathways were enriched in genes mutated only in EPCs. For the Ras-family genes, *KRAS* was mutated in only one EPC sample, while *HRAS* and *NRAS* mutations were relatively common in both EPCs and EPs. However, mutations were not detected in the hotspot regions of Ras-family genes. A recent study suggested that somatic mutations of *MAP2K1* activate the MAPK pathway in EPCs, and MAPK pathway alterations are a late event in EPC progression [18]. Our findings suggest that the Ras and MAPK signaling pathways may play a role in the development of EPCs.

Additionally, the PDGF signaling pathway was enriched in genes mutated only in EPCs. Multiple *PDGFRA* and *PDGFRB* mutations were found in exons 12 and 14, where recurrent mutations have been reported in other tumors [60–62]. One EPC harbored a p.R561C mutation, which is associated with activation of PDGFR-β signaling in familial myofibroma patients [63].

Mutations were found in multiple genes mutated in EPCs in the DPA sample. Such genes included *CDKN2A*, *ERBB4*, *NCOR1*, *PDGFRA*, and *PTCH1*. Interestingly, the sample showed a high weight on sign 1 in the mutation signature analysis, suggesting involvement of UV exposure. DPA is a rare malignant tumor originating from the eccrine sweat glands [64]. Human papillomavirus 42 integration and expression of HPV42 oncoproteins in DPAs have been reported [65]. Our results suggest that DPAs share some genetic features with EPCs and EPs.

Our study was limited by the small cohort size due to the rarity of EPCs. Such a cohort size often makes the detection of rare variants challenging [66]. Investigating the association between mutations and prognosis was not possible due to the small sample number. Furthermore, our study lacked healthy tissue controls for WES, thereby precluding the identification of germline and somatic variations from each other.

Overall, WES provided valuable insights into the general heterogeneity of EPCs and EPs, revealing at the same discrepancies between the two tumor types, such as exclusive mutations of *TP53*, *NCOR1*, *CDKN2A,* and *GSK3B* in EPCs and a higher mutational load in EPCs than in EPs. Additionally, we identified alterations in pathways associated with cell adhesion and extracellular matrix in EPCs, while pathways associated with ketone body and amino acid metabolism were altered in EPs. The MAPK and Ras signaling pathways were enriched in genes mutated only in EPCs. These findings elucidate the possible genetic mechanisms involved in the malignant transformation of EPs or the development of de novo EPCs. More evidence supporting the involvement of disrupted genes and pathways in tumorigenesis and the growth of EPCs, potentially through the utilization of cell lines or in vivo studies, is necessary to facilitate development of targeted therapeutic strategies and to increase understanding of this understudied tumor.

## Conclusions

EPCs and EPs are generally heterogeneous tumor entities, but there are also distinct discrepancies between them, such as exclusive *TP53*, *NCOR1*, and *CDKN2A* mutations in EPCs and a higher mutational load in EPCs than in EPs. Moreover, EPCs harbored alterations in pathways associated with cell adhesion and the extracellular matrix, in addition to the MAPK and Ras signaling pathways. The results revealed genes and signaling pathways that may play a role in EPC development. Validation of their involvement may provide novel treatment options for EPCs, which currently lack standardized therapy regimens after surgery.

## Supplementary Information

Additional file 1. The model with five de novo signatures showed the lowest Bayesian information criterion

Additional file 2. A list of oncogenes selected for the analyses. Corresponding Ensembl gene codes are shown on the right side

Additional file 3. A list of statistically significant terms in each database, p-values, and the genes associated with each term

## Data Availability

The data generated in this study are available from the Helsinki biobank (Project ID: HBP2018003) and the Finnish Clinical Biobank Tampere (pirha.fi/tampereen-biopankki, Project ID: BB2018-006) upon request. The codes used in this study are available on GitHub (https://github.com/Rare-Cancers-Research-Group/Porocarcinoma). All variants obtained from the WES analysis that passed filtering are documented in the text file formats uploaded to Zenodo (DOI: https://doi.org/10.5281/zenodo.10701230).

## Abbreviations

EPC: Eccrine porocarcinoma
EP: Eccrine poroma
DPA: Digital papillary adenocarcinoma
FFPE: Formalin-fixed paraffin-embedded
WES: Whole-exome sequencing
ECM: Extracellular matrix
SNV: Single nucleotide variant
SBS: Single-base substitutions
