# Supplementary figures and images for: Whole-exome sequencing identifies distinct genomic aberrations in eccrine porocarcinomas and poromas
Source: Orphanet J Rare Dis. 2025 Feb 13;20:70. doi: 10.1186/s13023-025-03586-7 (PMC11823087; doi:10.1186/s13023-025-03586-7)

# Bayesian Information Criterion

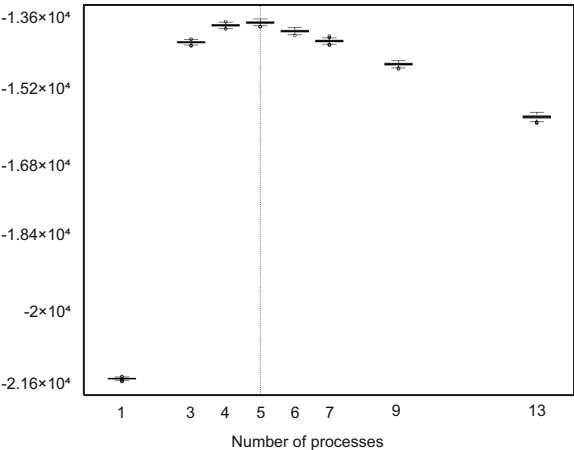

Supplement: Supplementary file 1 — Additional file 1. The model with five de novo signatures showed the lowest Bayesian information criterion [file 13023_2025_3586_MOESM1_ESM.pdf]
