# Supplementary material for: Whole-exome sequencing identifies distinct genomic aberrations in eccrine porocarcinomas and poromas
Source: Orphanet J Rare Dis. 2025 Feb 13;20:70. doi: 10.1186/s13023-025-03586-7 (PMC11823087; doi:10.1186/s13023-025-03586-7)
Supplement: Supplementary file 2 — Additional file 2. A list of oncogenes selected for the analyses. Corresponding Ensembl gene codes are shown on the right side [file 13023_2025_3586_MOESM2_ESM.pdf]

Additional file 2 Selected genes

Genes	Ensembl gene code
ABL1	ENSG00000097007
APC	ENSG00000134982
ARID1A	ENSG00000117713
ATM	ENSG00000149311
CACNA1S	ENSG00000081248
CDKN2A	ENSG00000147889
CSMD3	ENSG00000164796
EGFR	ENSG00000146648
ERBB2	ENSG00000141736
ERBB4	ENSG00000178568
FAT2	ENSG00000086570
GSK3B	ENSG00000082701
HRAS	ENSG00000174775
KMT2D	ENSG00000167548
KRAS	ENSG00000133703
LRP1B	ENSG00000168702
MET	ENSG00000105976
MUC16	ENSG00000291738
NCOR1	ENSG00000141027
NRAS	ENSG00000213281
PBRM1	ENSG00000163939
PDGFRA	ENSG00000134853
PIK3CA	ENSG00000121879
PTCH1	ENSG00000185920
PTEN	ENSG00000171862
RB1	ENSG00000139687
RET	ENSG00000165731
SETD2	ENSG00000181555
TP53	ENSG00000141510
TTN	ENSG00000155657
ZFHX4	ENSG00000091656
